# Effect of lipotoxicity on mitochondrial function and epigenetic programming during bovine in vitro embryo production

**DOI:** 10.1038/s41598-023-49184-0

**Published:** 2023-12-08

**Authors:** Ben Meulders, Waleed F. A. Marei, Inne Xhonneux, Peter E. J. Bols, Jo L. M. R. Leroy

**Affiliations:** 1 Laboratory of Veterinary Physiology and Biochemistry, Department of Veterinary Sciences, Gamete Research Centre, University of Antwerp, Wilrijk, Belgium; 2 Department of Theriogenology, Faculty of Veterinary Medicine, Cairo University, Giza, Egypt

**Keywords:** Developmental biology, Physiology

## Abstract

Maternal metabolic disorders may cause lipotoxic effects on the developing oocyte. Understanding the timing at which this might disrupt embryo epigenetic programming and how this is linked with mitochondrial dysfunction is crucial for improving assisted reproductive treatments, but has not been investigated before. Therefore, we used a bovine in vitro model to investigate if pathophysiological palmitic acid (PA) concentrations during in vitro oocyte maturation and in vitro embryo culture alter embryo epigenetic patterns (DNA methylation (5mC) and histone acetylation/methylation (H3K9ac/H3K9me2)) compared to control (CONT) and solvent control (SCONT), at the zygote and morula stage. Secondly, we investigated if these epigenetic alterations are associated with mitochondrial dysfunction and changes in ATP production rate, or altered expression of epigenetic regulatory genes. Compared to SCONT, H3K9ac and H3K9me2 levels were increased in PA-derived zygotes. Also, 5mC and H3K9me2 levels were increased in PA-exposed morulae compared to SCONT. This was associated with complete inhibition of glycolytic ATP production in oocytes, increased mitochondrial membrane potential and complete inhibition of glycolytic ATP production in 4-cell embryos and reduced SOD2 expression in PA-exposed zygotes and morulae. For the first time, epigenetic alterations in metabolically compromised zygotes and morulae have been observed in parallel with mitochondrial dysfunction in the same study.

## Introduction

In the past decades, the global prevalence of obesity has nearly tripled. Recent data from the World health organisation (WHO) reveals that 39% of adults are overweight (BMI > 25 and < 30) and 13% are obese (BMI > 30)^1^. Overweight or obese women of reproductive age have higher rates of infertility and pregnancy complications (e.g. gestational diabetes and miscarriage)^2,3^. The detrimental effects of maternal obesity are evident at the level of the oocyte and early embryo^4^. This was confirmed in a study where pregnancy rates were restored in obese women when donor oocytes from healthy mothers were used^5^.

Metabolic diseases, like obesity, often coincide with a state of upregulated lipolysis leading to increased serum non-esterified fatty acid (NEFA) concentrations. An increased availability of NEFAs can result in higher uptake in non-adipose tissues, which may lead to peripheral insulin resistance and pancreatic β cell dysfunction. This is called *lipotoxicity*^6^. Elevated serum NEFA concentrations are also reflected in the follicular fluid (FF) and oviductal fluid (OF) in cows and humans^7–9^ and have a detrimental impact on oocyte and embryo quality^10^. The saturated palmitic acid (PA) is the most abundant NEFA in human FF^11^ and was the only NEFA in the FF that is negatively correlated with pregnancy rates following intracytoplasmic sperm injection (ICSI)^12^. When PA is directly added to the maturation or culture medium at pathophysiological concentrations, bovine oocyte and embryo development are significantly hampered^13–15^. The increased uptake of NEFAs during oocyte maturation disrupts oocyte metabolism, causing increased mitochondrial (MT) activity^16^. This results in increased production of reactive oxygen species (ROS) which may lead to oxidative stress and as a consequence induce MT dysfunction^2,17^. In oocytes, MT dysfunction does not result in increased mitophagy^18^. Because of this, damaged mitochondria may accumulate in the oocyte and MT dysfunction may persist during early embryo development^19^. Further exposure of bovine embryos to lipotoxicity due to high concentrations of NEFAs in the OF may further exaggerate this impact^20,21^.

Exposure of bovine and porcine cumulus-oocyte-complexes (COCs) or bovine embryos to elevated concentrations of NEFAs has been shown to alter DNA methylation patterns in the produced blastocysts^21,22^. However, it’s not known if such epigenetic alteration occurs only during de novo methylation, or starts during earlier stages of embryo development. Epigenetic reprogramming during final oocyte maturation and pre-implantation development is a highly dynamic process which is strictly regulated by epigenetic regulatory enzymes that catalyse DNA and histone de-/remethylation and histone de-/reacetylation. A previous study showed that PA-exposure during in vitro maturation (IVM) alters mRNA expression of such an enzyme (DNMT3a) in cumulus cells^13^. This might be explained by PA-induced alteration of epigenetic patterns in this gene and/or altered availability of the required substrates^23^. DNA methylation and histone modification levels in the oocyte are high until the end of meiotic maturation just before ovulation, while histone acetylation is removed^24^. DNA is demethylated after fertilization, with mainly active, faster demethylation by TET enzymes of the paternal genome and mainly passive, slower demethylation of the female genome^25^. This continues until the blastocyst stage after which methylation levels are re-established by DNMT enzymes^26^. Histone acetylation and methylation follow more or less the same dynamics during early embryo development, with specific differences related to the class, residues, and modification-valency of core histones^27^. The importance of the epigenetic reprogramming during early embryo development is to acquire totipotent cells to ensure proper development, and erase epigenetic modifications that accumulated in the parents to avoid detrimental offspring effects^28^. As a consequence, improper establishment or erasure of epigenetic marks might be irreversible and may lead to permanently altered gene expression, abnormal development and early onset of disease^29,30^. Understanding the timing at which epigenetic programming might be disrupted due to oocyte exposure to lipotoxicity (as in oocytes collected from obese women in human in vitro fertilization (IVF) clinics) is crucial for assisted reproductive treatment (ART) application. For example, development of in vitro embryo therapy that can normalise epigenetic programming during culture and safeguard offspring health requires fundamental insight to define the optimal intervention time and target mechanisms.

In somatic cells, it has been shown that epigenetic programming is dependent on MT functions^31^. MT ATP plays an important role in the activation and biosynthesis of epigenetic regulatory enzymes, intermediates and methyl donors that influence nuclear epigenetic programming^32,33^. In contrast, such mito-nuclear crosstalk is not well defined in oocytes. Metabolic alterations that impact MT function in oocytes may lead to epigenetic alterations during subsequent early embryo development^34^. However, this has not been clearly demonstrated. Association of alterations in epigenetic programming with MT function and ATP production rates has not been previously illustrated in metabolically compromised oocytes or embryos. On the other hand, apart from the impact on MT functions, lipotoxicity is known to alter the expression of regulatory genes involved in methylation and acetylation processes^35,36^ as well as in blastocysts upon direct exposure to high concentrations of NEFAs^21^. This was not examined during earlier stages of development.

Therefore, we hypothesise that oocyte maturation and embryo development in a lipotoxic microenvironment can disturb DNA and histone epigenetic marks already during very early stages of development (at the zygote and morula stage), and that this is associated with MT bioenergetic dysfunction and/or altered expression of epigenetic regulatory genes. We aimed to test this hypothesis by exposing bovine COCs and early embryos to pathophysiologically relevant concentrations of PA during IVM and in vitro culture (IVC) as a model to mimic the lipotoxic FF and OF microenvironment in mothers suffering from metabolic disorders. DNA methylation and histone modification marks, MT function and expression of epigenetic regulatory genes and genes related to oxidative stress were assessed during early embryo development. Also, MT function was assessed in oocytes. Understanding the patterns and mechanisms by which epigenetic alterations may develop during early embryo development may assist in understanding the potential long term consequences on fetal development and postnatal health and in developing strategies to prevent or alleviate these defects.

## Results

### Developmental competence

PA-exposure during IVM and IVC resulted in a significant decrease in cleavage rate compared to both the standard laboratory control (CONT) and the solvent control (SCONT) (*P* < 0.001). The percentage of 4-cell embryos was also decreased in the PA group compared to both controls (*P* < 0.05), while the percentage of fragmented embryos was not significantly different between groups (Table 1). The day 4.7 morula rate was significantly decreased in the PA group compared to CONT and SCONT (*P* < 0.001) (Table 2). For blastocyst rates, on day 7 and 8 there was a significant decrease in the PA group compared to both controls (*P* < 0.01). Blastocyst rates were also calculated as a percentage of the cleaved embryos. On day 7 and 8, this was significantly decreased in PA compared to both controls (*P* < 0.05) (Table 1). For none of these parameters there was a significant difference between the two controls confirming that the solvent had no effect on development (Tables 1, 2).

**Table 1:** Effect of PA-exposure during IVM and IVC on embryo cleavage (at 48 h post fertilisation) and blastocyst rates (at day 7 and 8).

	CONT	SCONT	PA
Cleavage			
Total COCs (n)	267	272	266
Cleaved embryos (% from total)	221 (82.8%)^a^	224 (82.4%)^a^	182 (68.4%)^b^
4-cell stage or more (% from total)	175 (65.6%)^a^	173 (63.6%)^a^	91 (34.2%)^b^
Fragmented embryos (% from total)	24 (9.0%)^a^	26 (9.6%)^a^	46 (17.3%)^a^
Blastocysts			
Total COCs (n)	173	176	171
D7 blastocysts (% from total)	58 (33.6%)^a^	58 (33.0%)^a^	27 (15.8%)^b^
D7 blastocysts (% from cleaved)	58 (39.3%)^a^	58 (41.6%)^a^	27 (22.3%)^b^
D8 blastocysts (% from total)	72 (41.6%)^a^	69 (39.2%)^a^	43 (25.1%)^b^
D8 blastocysts (% from cleaved)	72 (49.8%)^a^	69 (49.4%)^a^	43 (35.8%)^b^

Data are presented as total and proportions (%). Significant differences (*P* < 0.05) are shown by different superscripts (a or b). In total 4 replicates were performed.

*CONT* Control, *SCONT* Solvent control, *PA* Palmitic acid, *COC* Cumulus-oocyte complex.

**Table 2:** Effect of PA-exposure during IVM and IVC on morula rate at day 4.7.

	CONT	SCONT	PA
Total embryos (n)	1370	1364	2599
D4.7 morulae (% from total)	194 (14.2%)^a^	184 (13.5%)^a^	138 (5.3%)^b^

Data are presented as total and proportions (%). Significant differences (*P* < 0.05) are shown by different superscripts (a or b). In total 16 replicates were performed.

*CONT* Control, *SCONT* Solvent control, *PA* Palmitic acid.

### Global DNA methylation

**Figure 1:**
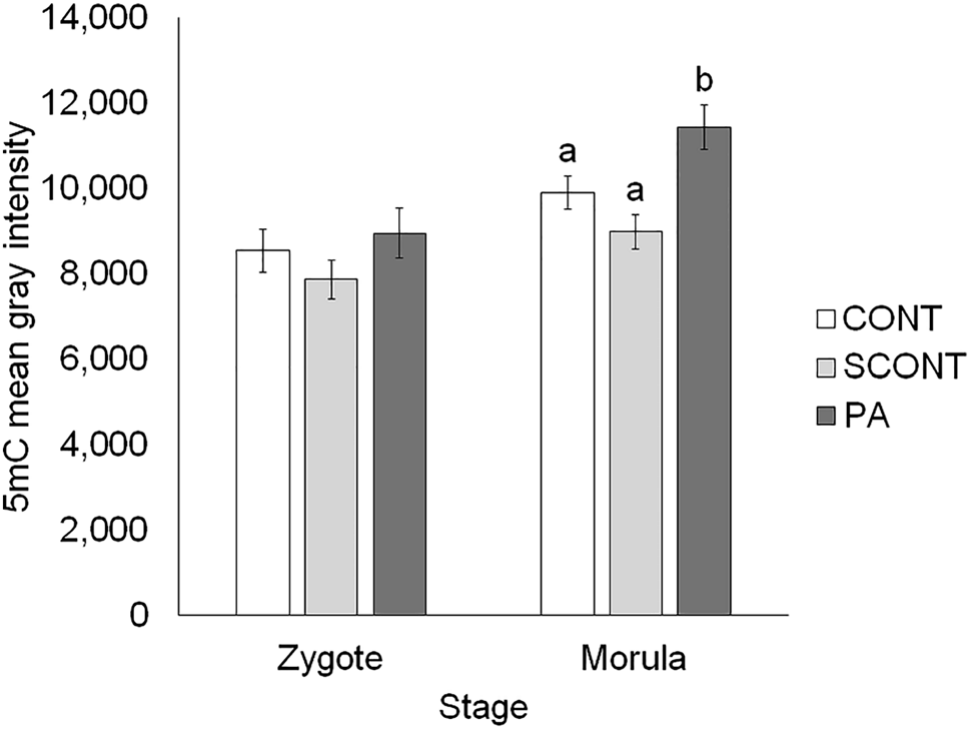
Quantification of 5mC immunostaining as an estimate of global DNA methylation levels in zygotes and morulae. Each bar shows mean ± SEM. Significant differences (*P* < 0.05) are shown by different letters (a or b). Created with BioRender.com.

In zygotes, global 5mC levels were not affected by solvent or PA-exposure (*P* > 0.1). However, in morulae global 5mC levels were increased in PA compared to CONT (*P* = 0.017) and SCONT (*P* < 0.001) (Fig. 1). See Supplementary Figs. S1 and S2 online for representative images.

### Histone modifications

**Figure 2:**
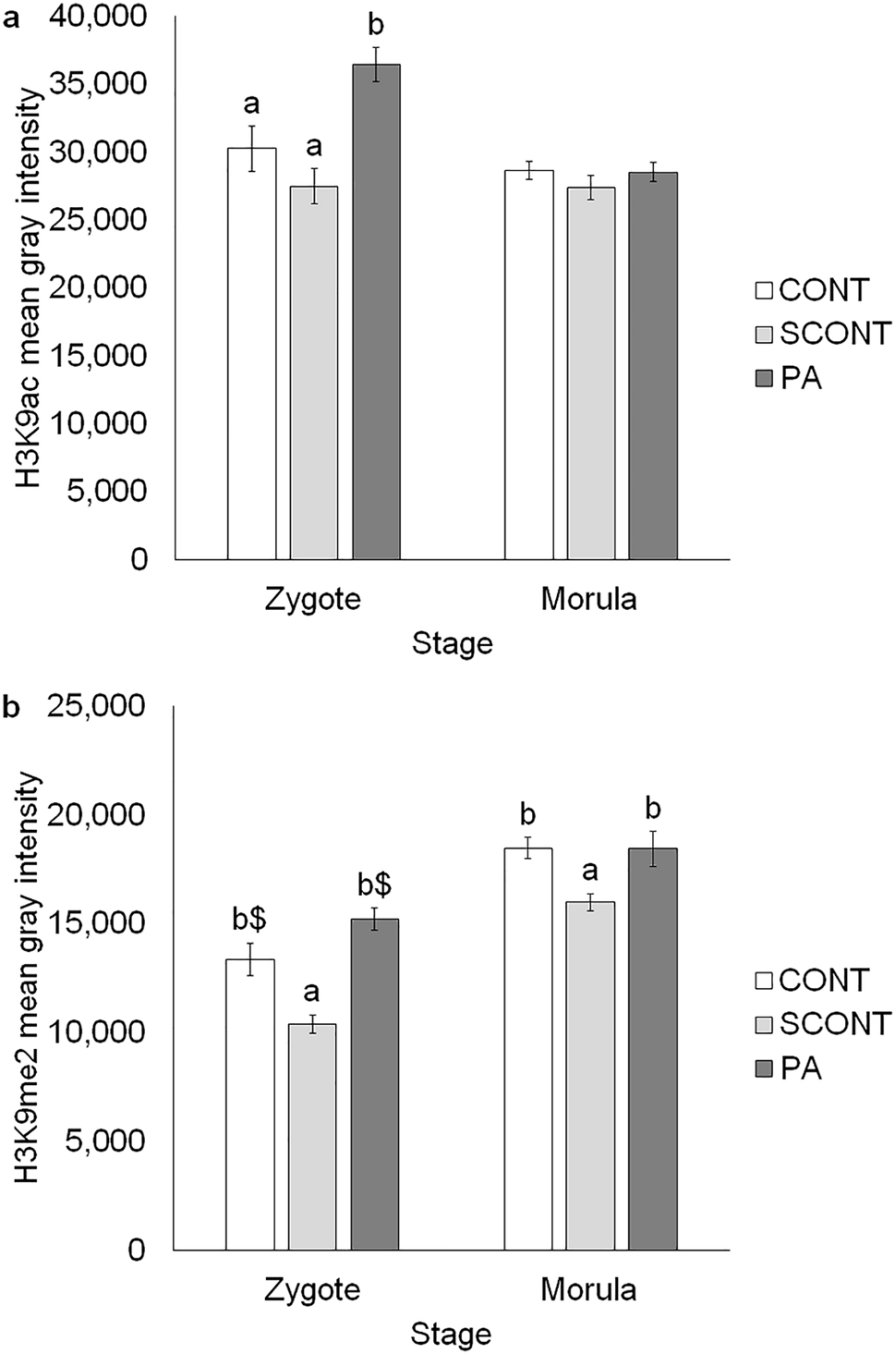
(**a**) Quantification of H3K9ac immunostaining as an estimate of histone acetylation levels in zygotes and morulae. (**b**) Quantification of H3K9me2 immunostaining as an estimate of histone methylation levels in zygotes and morulae. Each bar shows mean +/− SEM. Significant differences (*P* < 0.05) are shown by different letters (a, b or c), tendencies (*P* < 0.1 and *P* > 0.05) are shown by a letter with a dollar sign ($).

In zygotes, H3K9ac levels were increased in PA compared to CONT (*P* = 0.007) and SCONT (*P* < 0.001). In morulae however, no effect of PA was observed (*P* > 0.1) (Fig. 2a).

PA significantly increased H3K9me2 levels in zygotes (*P* < 0.001) and morulae (*P* = 0.039) compared to SCONT. Nevertheless, contrary to what was seen in 5mC and H3K9ac, the addition of ethanol (0.1%) significantly reduced H3K9me2 levels compared to CONT in zygotes (*P* = 0.011) and morulae (*P* = 0.001). This apparently also influenced the PA effects since there was only a tendency for increase compared to CONT in zygotes (*P* = 0.085) and no difference in morulae (*P* > 0.1) (Fig. 2b). See Supplementary Figs. S1 and S2 online for representative images.

### ATP production rate and MT activity

#### MII oocytes

**Figure 3:**
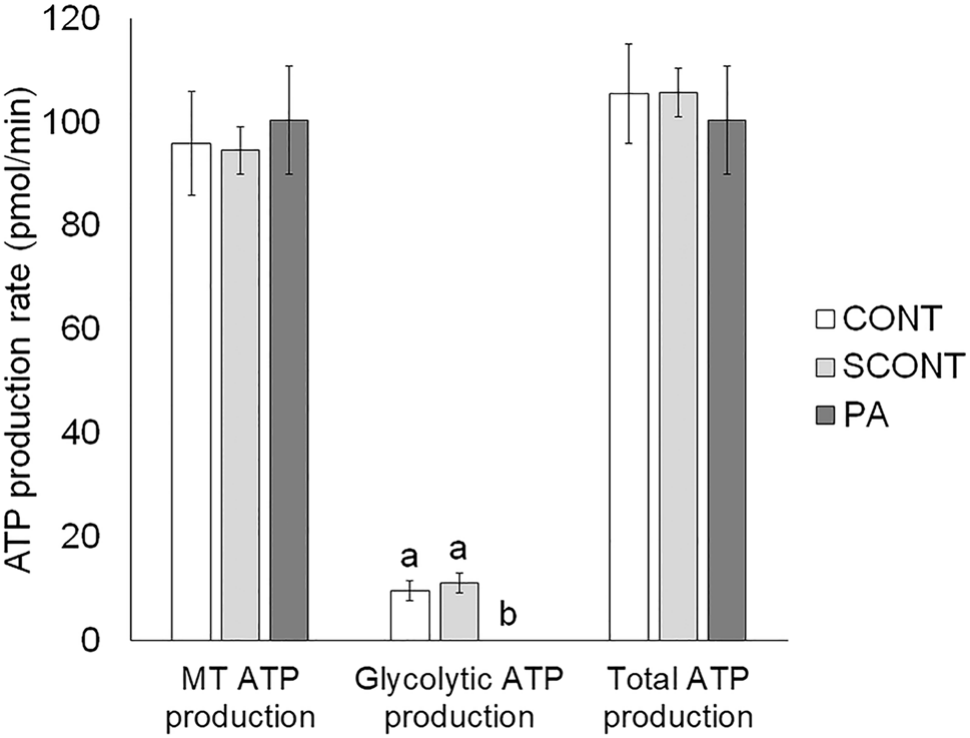
Quantification of mitochondrial, glycolytic and total ATP production rate using the Seahorse XF Mini Bioanalyzer in oocytes at 24 h of IVM. Data are presented as mean ± SEM. Significant differences (*P* < 0.05) are shown by different letters (a or b).

No significant differences in total or MT ATP production were observed between the groups (*P* > 0.1). Interestingly, glycolytic ATP production was completely inhibited in PA-exposed oocytes compared to CONT (*P* = 0.015) and SCONT (*P* = 0.005) (Fig. 3).

#### 4-Cell embryos

**Figure 4:**
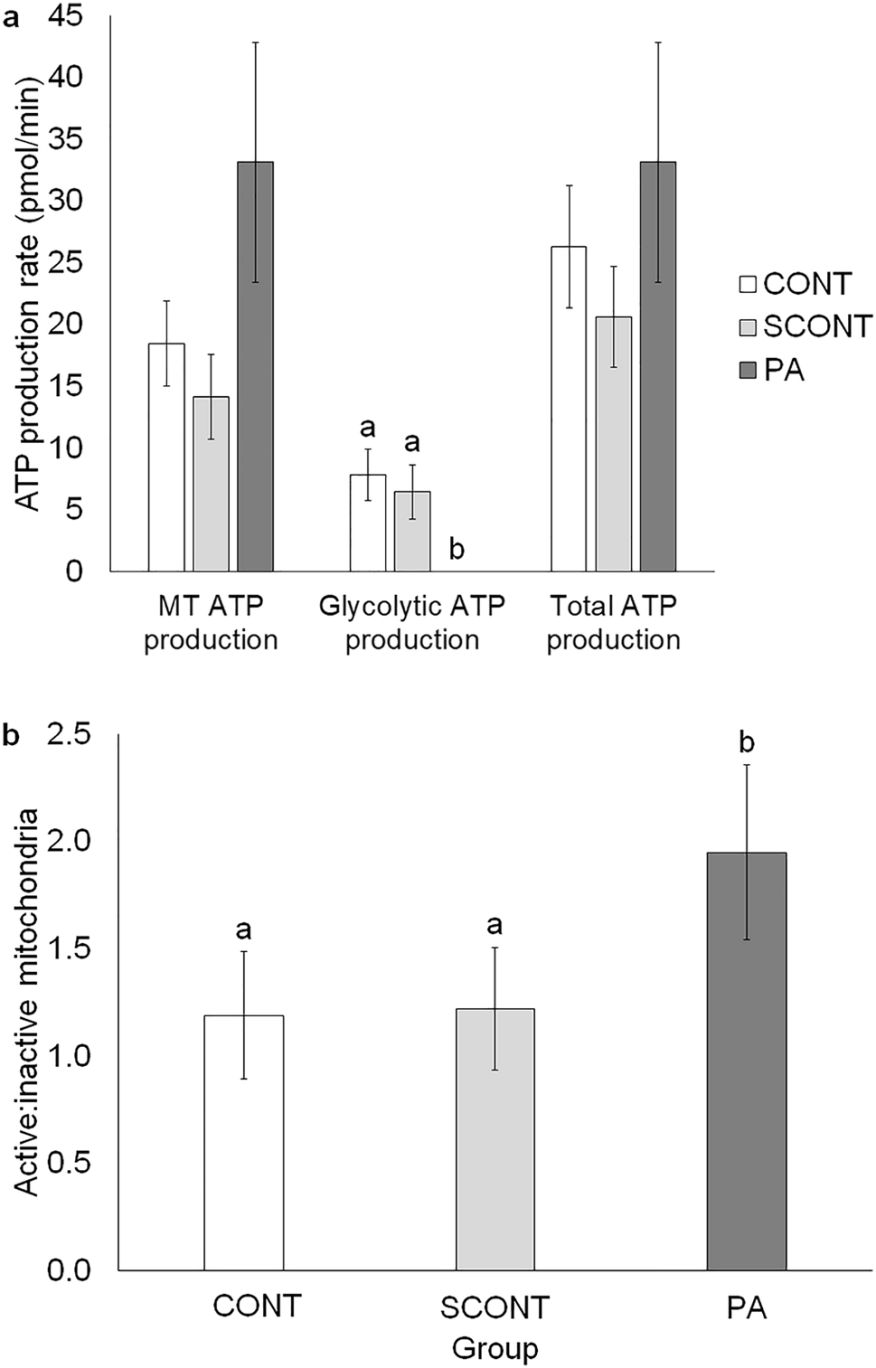
(**a**) Quantification of mitochondrial, glycolytic and total ATP production rate using the Seahorse XF Mini Bioanalyzer in 4-cell embryos. Data are presented as mean +/− SEM. Significant differences (*P* < 0.05) are shown by different letters (a or b). (**b**) Quantification of mitochondrial membrane potential (MMP) by calculating the ratio of j-aggregates (active mitochondria) to monomers (inactive mitochondria) using JC-1 staining in 4-cell embryos. Data are presented as mean +/− SEM. Significant differences (*P* < 0.05) are shown by different letters (a or b).

No significant differences in MT or total ATP production were observed in 4-cell embryos between the groups (*P* > 0.1). However, as observed in the oocytes, glycolytic ATP production was completely inhibited in PA-exposed 4-cell embryos compared to CONT (*P* = 0.009) and SCONT (*P* = 0.043) (Fig. 4a). The ratio of j-aggregates to monomers is a measure for MT membrane potential (MMP). Here, this ratio was significantly increased in PA-exposed 4-cell embryos compared to CONT (*P* < 0.001) and SCONT (*P* < 0.001) (Fig. 4b). See Supplementary Fig. S3 online for representative images.

### mRNA expression of MT and epigenetic regulatory genes

**Figure 5:**
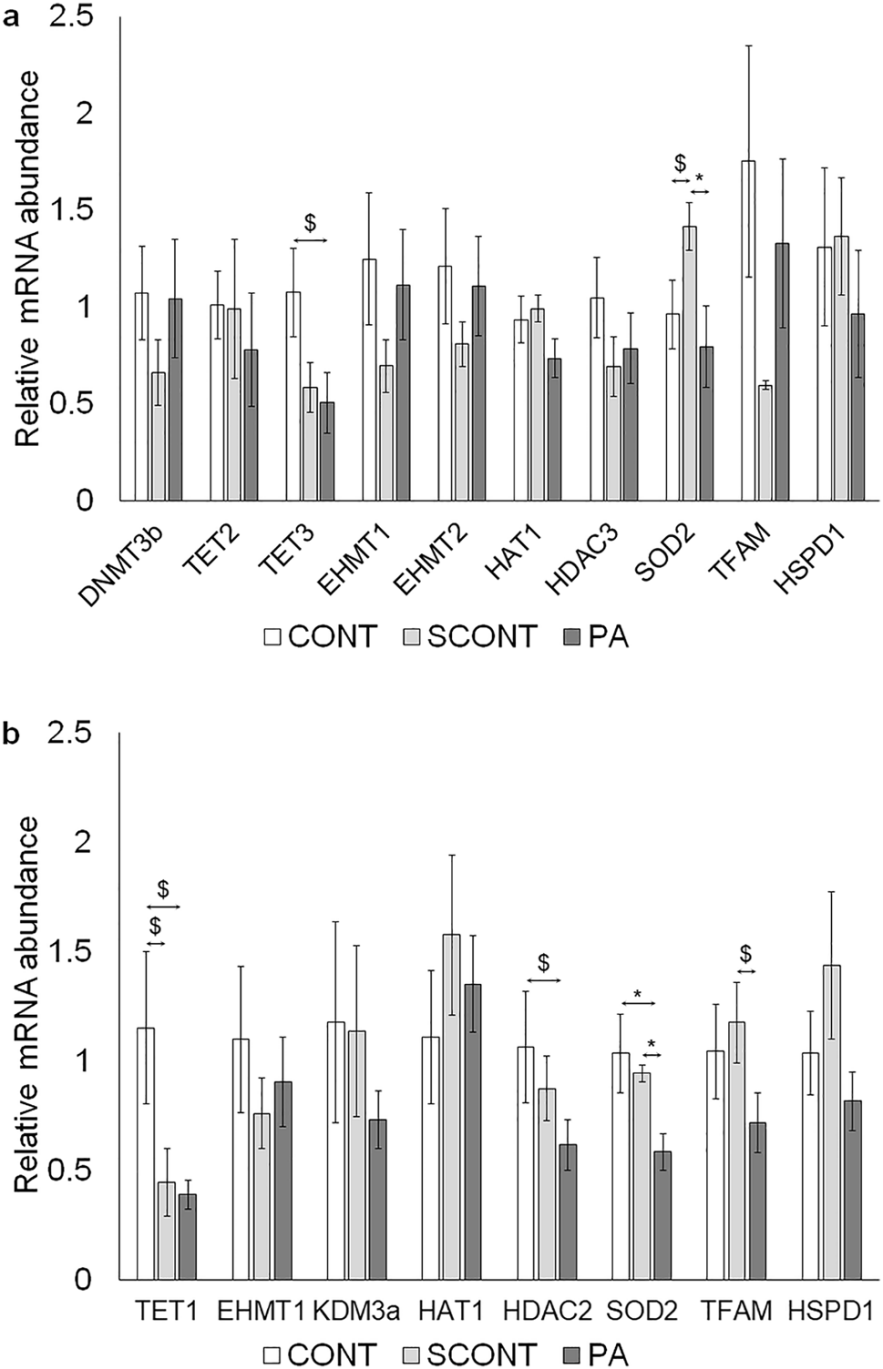
(**a**) Quantification of mRNA transcript abundance in zygotes of epigenetic regulatory genes (DNMT3b, TET2, TET3, EHMT1, EHMT2, HAT1, HDAC3), genes involved in regulation of oxidative stress (SOD2) and genes that are important for MT function (TFAM, HSPD1). (**b**) Effect of PA-exposure during IVM and IVC on mRNA transcript abundance in morulae of epigenetic regulatory genes (TET1, EHMT1, KDM3a, HAT1, HDAC2), genes involved in regulation of oxidative stress (SOD2) and genes that are important for MT function (TFAM, HSPD1). Each bar shows mean +/− SEM of fold changes relative to expression level of housekeeping genes (18S & YWHAZ). Significant differences (*P* < 0.05) are shown by an asterisk (*), tendencies (*P* < 0.1 and *P* > 0.05) are shown by a dollar sign ($).

At the zygote stage, no major differences in mRNA transcript abundance were seen in genes involved in epigenetic regulation between the groups. For TET3, there was a tendency for downregulation in PA compared to CONT (*P* = 0.064). Also, the expression of SOD2 was significantly downregulated in PA zygotes compared to SCONT (*P* = 0.026) and had a tendency for downregulation in CONT compared to SCONT (*P* = 0.088). For all other genes, no differences were seen between any of the groups (*P* > 0.1) (Fig. 5a).

Similarly in morulae, no major differences in mRNA transcript abundance were seen in genes involved in epigenetic regulation between the groups. Both PA and solvent resulted in a tendency for a lower TET1 expression compared to CONT (*P* = 0.077 and 0.097 respectively). Also, expression of HDAC2 had a tendency for downregulation in PA compared to CONT (*P* = 0.097). PA-exposure during IVM and IVC resulted in a significantly downregulated expression of SOD2 compared to CONT and SCONT (*P* = 0.015 and *P* = 0.029 respectively). Finally, TFAM expression had a tendency for downregulation in PA compared to SCONT (*P* = 0.092). For all other genes, no differences were seen between the groups (*P* > 0.1) (Fig. 5b).

## Discussion

The aim of this study was to examine if in vitro PA-induced lipotoxicity during bovine oocyte maturation and early embryo development can alter DNA and histone epigenetic marks and at which embryonic stage this might be detected. Secondly, we aimed to investigate if these alterations can be linked with changes in MT functions and/or expression of epigenetic regulatory genes. The results show that PA-exposure during final oocyte maturation altered histone modifications in zygotes and continued exposure during early embryo development resulted in altered DNA and histone methylation in morulae. This was associated with changes in oocyte ATP production and altered MT function and ATP production in 4-cell embryos, but could not be explained by changes in regulatory gene expression.

Cleavage, morula and blastocyst rates were significantly reduced in the PA group compared to both controls. This supports earlier research where the same was observed after NEFA-exposure during IVM or IVC only^37,38^. However, comparing these results with studies where a mixture of different NEFAs were used and/or NEFA-exposure was only done during IVM or IVC should be done with caution.

Our results showed that global DNA methylation levels were not altered in the zygotes due to PA-exposure during IVM and IVC. Previous studies have shown that exposure of bovine COCs to PA (150 µM) during IVM resulted in a significant increase in MMP of mature oocytes^38^. MT activity is known to influence epigenetic programming through providing ATP, which is necessary for the activity of S-adenosylmethionine methyltransferase and the production of SAM, the methyl donor for DNA and histone methylation^39^. DNA methylation levels are also known to significantly increase during oocyte maturation^24^. Therefore, we expected that an increase in oocyte MT activity due to PA-exposure should increase DNA methylation levels already at the zygote stage, but this was not detected. We also found that PA completely inhibited glycolytic activity in oocytes and numerically increased MT ATP production rate. This is in accordance with the previously reported increase in MMP^38^ and may suggest a subtle increase in dependence on fatty acid β-oxidation for energy production. Oocytes are known to rely on oxidative phosphorylation (OXPHOS) for ATP production, with minimal glycolytic activity^40^. The overall ATP production rate was however not affected by PA. There were also no PA-dependent differences in expression of genes related to de novo DNA methylation or DNA demethylation in zygotes. It’s important to note that gene expression has been shown to differ between male and female embryos produced under in vitro conditions^41^. In this experiment, we did not take embryo sex into account. This might increase variation in our results and lead to the absence of group effects. Nevertheless, the absence of detectable changes in global DNA methylation in PA-derived zygotes does not rule out possible local DNA methylation changes in specific genes that cannot be detected with immunostaining.

As the exposure to PA continued, PA-exposed morulae exhibited a significant increase in global DNA methylation levels. This was also reported in blastocysts after exposure to pathophysiological concentrations of NEFAs during IVM or IVC only^21,22^, meaning that these alterations may already be initiated at earlier stages. We also monitored MT activity in 4-cell embryos. PA-exposed 4-cell embryos had a significant increase in MMP and a numerical increase in MT ATP production rate. As mentioned above, such increase in bioenergetic activity may mediate, at least in part, the increase in DNA methylation. The increase in MT MMP might be associated with alterations in the concentration of tricarboxylic acid (TCA) metabolites that can also affect the epigenome. For example, succinate and α-ketoglutarate (α-KG) inhibit and stimulate TET activity, respectively^33,42^. If succinate levels increase and/or α-KG levels drop, this will result in higher global DNA methylation levels after the demethylation wave since TET is inhibited. This might explain the increased global DNA methylation levels in PA-exposed morulae. Another explanation might be a decreased activity of DNA demethylation enzymes or increased activity of de novo DNA methylation enzymes between the zygote and morula stage. However, we did not see any differences in expression of DNMT and TET enzymes in zygotes and morulae when comparing PA to SCONT, at the transcriptomic level. This does not rule out possible alteration in their activity which was not examined in this study.

On the other hand, histone modifications also play a very crucial role in epigenetic regulation especially during early embryo development^43^. We showed that a significant increase in global histone acetylation and methylation levels was already detectable in the zygotes after exposure to PA during IVM. In fact, the histone modification alterations at this stage might explain the alterations in DNA methylation levels in the morulae. For example, histone modifications can alter the accessibility of DNA to DNMT enzymes which ultimately results in DNA methylation alterations^44,45^. We tried to explain this by measuring glycolytic ATP production rate in oocytes, which was reduced while total ATP production rate was not altered. This means that the cell is relying more on OXPHOS for ATP production. Increased OXPHOS activity might lead to increased production of TCA metabolites such as citrate. This can be converted to acetyl-coA, which can then be used as acetyl donor for histone acetylation^33^. Also, increased TCA cycle activity will lead to depletion of NAD + , which is a substrate of histone deacetylases, leading to increased histone acetylation levels^46^. Also, somatic cell studies have shown that metabolites of β-oxidation can block glucose metabolism^47^. More recent research has shown that inhibition of β-oxidation during murine IVM results in increased glucose uptake by COCs^48^, and that blastocysts from obese women and mice display decreased rates of glucose uptake^49,50^. As a consequence, oocytes and embryos will favour oxidation of fatty acids as an energy source in a lipotoxic environment, which will supply more acetyl-coA compared to glycolysis^51^. All this could explain the increased H3K9ac level in PA-exposed zygotes. However, it’s important to consider is that there are other sources of acetyl-coA, like amino acids. So drawing conclusions about acetyl-coA levels is difficult based on these data. The increased histone methylation levels might result from increased TCA cycle activity that is coupled with the increased OXPHOS. Similar to what was described for DNA methylation, α-KG and succinate respectively stimulate and inhibit HDMs, which can lead to increased histone methylation levels when α-KG is decreased or succinate is increased^33,52^. Again, we also tried to explain the epigenetic changes by altered expression of genes that are responsible for de novo histone acetylation/methylation or histone deacetylation/demethylation. But we didn’t see any differences between the treatment groups.

In contrast to what was observed in zygotes, histone acetylation in morulae was not different in PA-exposed embryos. This may thus imply that after fertilisation, histone deacetylation was increased or de novo histone acetylation was decreased due to PA-exposure during IVC, bringing it back to the level of the control groups. However, no PA-dependent differences in expression of genes related to de novo histone acetylation or histone deacetylation in zygotes or morulae were observed. Finally, in morulae increased histone methylation was seen due to PA-exposure. As explained before, this might be caused by changes in the availability of MT metabolites. The increased MMP in 4-cell embryos was associated with numerically increased MT ATP production rate without altering total ATP production rate in 4-cell embryos. This might alter concentrations of TCA metabolites, affecting the epigenome. Identical to what was already described for histone methylation in zygotes, α-KG and succinate respectively stimulate and inhibit HDMs, which can lead to increased histone methylation levels when α-KG is decreased or succinate is increased^33,52^. Another explanation for the increased histone methylation might be a decreased expression of histone demethylation genes or increased expression of de novo histone methylation genes during early embryo development. However, we did not see any differences in expression of these genes in zygotes or morulae.

Optimal levels of DNA methylation and histone modifications during oocyte maturation and early embryo development are required for key biological processes, such as cell differentiation, embryonic genome activation and genomic imprinting^53^. The dynamic properties of the epigenetic landscape during oocyte maturation and early embryo development makes this a highly vulnerable window for disruption of epigenetic programming due to environmental changes^54^. Our results show that 5mC levels increase with 13.5% and 21.5% in PA-exposed morulae compared to CONT and SCONT, respectively, and H3K9ac increases with 16.9% and 24.5% in PA-exposed zygotes compared to CONT and SCONT, respectively. Also, H3K9me2 increases with 31.7% and 13.4% in PA-exposed zygotes and morulae, respectively, compared to SCONT. This can have potential functional consequences. Firstly, as DNA methylation is correlated with silencing of gene transcription^55^, the increased DNA methylation levels at the morula stage due to PA might interrupt cell differentiation during gastrulation later in development^56^. Also, acetylation of lysine 9 on H3 in the promotor region is associated with gene activation^57^. Similar to DNA methylation, histone H3 Lysine 9 dimethylation (H3K9me2) is associated with silencing of gene transcription^27,58^. At the zygote stage, H3K9me2 is important for replacement of protamines and proper chromosomal segregation during cell division^59^. Additionally, H3K9me2 can also play a role in imprinting, repression of transposable elements and zygotic genome activation^60–62^. Histone methylation at the morula stage is required for regulation of embryonic genome activation, X-chromosome inactivation and chromatin reorganization^43,63^.

To further increase the understanding of the epigenetic alterations in early embryos, we also assessed the mRNA transcript abundance of genes related to MT function and oxidative stress. In zygotes and morulae, SOD2 (marker of anti-oxidative activity) mRNA transcript abundance was significantly decreased in the PA group compared to SCONT (and also compared to CONT in morulae). Usually, this is interpreted as a lower transcriptional activity of this gene. It’s however important to consider that in cattle embryonic genome activation (EGA) is initiated at the 4-cell stage, followed by a major EGA at the 8-cell stage^64^. Before this, no active mRNA transcription occurs. Instead, early development is regulated by post-transcriptional modifications, translation or degradation of maternally inherited mRNA^65^. This is supported by the fact that SOD2 transcript abundance decreases from the MII oocyte to the morula stage^66^. In that case, downregulation can be interpreted as an increased need for this gene’s product. The decreased SOD2 mRNA abundance might thus suggest that oxidative stress is increased in PA-exposed zygotes and morulae. Furthermore, there was a tendency for a decreased expression of TFAM (marker of MT biogenesis) in PA-exposed morulae compared to SCONT. This may imply that MT biogenesis is stimulated, mostly as a response to MT damage. However, previous research has shown that MT biogenesis is not active until the blastocyst stage^67^. For HSPD1 (marker of MT UPR), no difference between any of the groups was seen in zygotes nor morulae. This implies that PA-exposure during oocyte and embryo development does not affect MT protein folding.

Finally, the effect of ethanol as a solvent is also important to mention. Previous research has shown that addition of 0.1% ethanol during bovine IVM increases oocyte MMP and H3K9me3 levels in 8-cell embryos^68^. We only observed a significant effect of SCONT compared to CONT in H3K9me2 levels in zygotes and morulae and in SOD2 and TET1 mRNA abundance in zygotes and morulae, respectively. This confirms the importance of choosing the right reference group as a control.

## Conclusions

In conclusion, we have shown for the first time that zygotes derived from metabolically compromised oocytes (matured under lipotoxic conditions) already exhibit increased histone acetylation and methylation levels without a significant change in global DNA methylation yet. With continued exposure to a lipotoxic microenvironment during early embryo development, DNA and histone methylation marks were increased in morulae. We showed possible associations with oocyte and embryo ATP production and embryo MT dysfunction, while there was no link observed with mRNA expression of epigenetic regulatory genes. Future research might focus on determining concentrations of MT metabolites, such as SAM and acetyl-coA, to correlate this with the observed epigenetic alterations. These fundamental insights can help in developing ART applications to safeguard epigenetic programming and offspring health in obese individuals. Such interventions should aim at supporting MT functions already during oocyte maturation and during the early stages of embryo cleavage. Ongoing research in our laboratory further investigates the causal link between MT dysfunction and epigenetic alterations in oocytes and early embryos.

## Materials and methods

### Material

All chemicals were purchased from Sigma Aldrich (Overijse, Belgium) unless otherwise stated.

### Experimental design

Good quality immature bovine COCs (homogenous, dark ooplasm and > 5 compact layers of cumulus cells) were matured in vitro in one of three conditions: CONT, SCONT and PA. In the PA group, a pathophysiological concentration of 150 µM PA was used; as measured in the follicular micro-environment in obese women^7^ and cows during negative energy balance^8^. To focus on oocyte/embryo-born effects and avoid indirect effects through alterations in sperm functions, IVF was done under standard fatty acid-free conditions. During IVC, a concentration of 230 µM PA was used in the PA group. This resembles the serum and oviductal fluid pathophysiological concentrations^9^. Exposure to a lipotoxic insult was performed during IVM and IVC as a model that is pathophysiological more relevant compared to exposing only during IVM or IVC, since in obese individuals the lipotoxic effect occurs in both the ovary and reproductive tract. Furthermore, the only NEFA that was added was PA as this is the most abundant NEFA in FF^11^ and is also the only NEFA in the FF that was retrospectively negatively correlated with pregnancy success rates following ICSI in women^12^.

**Figure 6:**
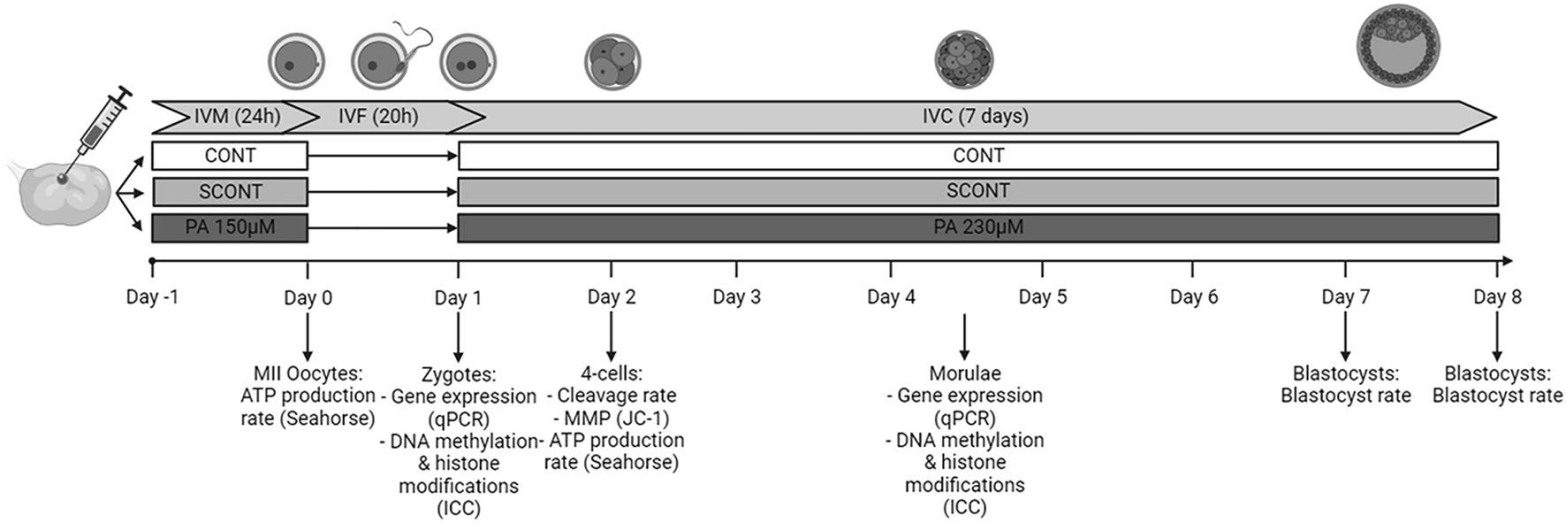
Overview of the experimental design. Bovine cumulus-oocyte complexes and embryos were exposed to control (CONT), solvent control (SCONT) or palmitic acid (PA) during in vitro maturation (IVM) and in vitro culture (IVC). *IVF* In vitro fertilisation, *ICC* Immunocytochemistry, *qPCR* Quantitative polymerase chain reaction, *JC-1* 5,5′,6,6′-tetrachloro-1,1′,3,3′-tetraethyl-benzimidazolyl-carbocyanine iodide, *MMP* Mitochondrial membrane potential.

In the results section, the experiments are described in the same sequence of the hypotheses and research questions described above. After reporting the effect on developmental competence as a validation of the model, we started with the main effects on epigenetic modulation, followed by looking into the mechanisms by which these effects might be mediated. We firstly examined if PA-exposure during IVM and IVC has an effect on DNA methylation in zygotes (3 replicates, 26–30 embryo’s/group) and morulae (3 replicates, 18–22 embryo’s/group). Then, the same was done for histone methylation/acetylation in zygotes (3 replicates, 26–30 embryo’s/group) and morulae (5 replicates, 34–40 embryo’s/group). Since PA was found to alter epigenetic patterns in zygotes and morulae, we further tested potential associations with MT MMP in 4-cell embryos (3 replicates, 16–18 embryos/group), and ATP production rate of MII oocytes (6 replicates; pools of 14–20 oocytes/group) and 4-cell embryos (5 replicates; pools of 10–22 embryos/group). To show if PA-induced alteration in epigenetic patterns are mediated by transcriptomic alterations, we examined the gene expression of marker genes involved in epigenetic regulation, MT function and oxidative stress in zygotes (5–8 replicates; pools of 10–20 zygotes/group/replicate) and morulae (4 replicates; pools of 7–30 zygotes/group/replicate). Developmental competence was assessed by recoding embryo cleavage and blastocyst rates (4 replicates), and morula rates (16 replicates) (Fig. 6).

### Collection and selection of bovine COCs

Bovine ovaries were collected from a local abattoir in warm saline solution (NaCl 0.9%) and transferred to our laboratory within 1-3 h of slaughter. Then, they were washed twice in warm NaCl 0.9% supplemented with kanamycin (125 µg/ml). Antral follicles with a diameter of 2-6 mm were aspirated using an 18G needle affixed to a 10 ml syringe. The aspirated FF was emptied in 15 ml tubes which were centrifuged for 1 min at 13 g. The cellular precipitate was then transferred to a 90 mm petri dish containing sterile Hepes-buffered Tyrode’s albumin lactate pyruvate media (Wash-TALP). Unexpanded COCs with dark homogeneous cytoplasm surrounded by five or more cumulus cell layers (quality grade I and II) were selected under an Olympus SZX7 stereomicroscope (+/− 98 COCs/group/replicate)^69^.

### In vitro maturation (IVM)

Maturation medium was composed of TCM-199 medium supplemented with L-glutamine (0.4 mM), sodium pyruvate (0.2 mM), gentamicin (50 µg/ml), cysteamine (0.1 µM), murine epidermal growth factor (mEGF, 20 ng/ml) and fatty acid-free bovine serum albumin (BSA) (0.75% w/v) as a carrier of PA. COCs were washed in Wash-TALP and transferred to equilibrated four-well plates in groups of 50–60 COCs (10 µl maturation medium/COC). In the PA group, PA was added at a concentration of 150 µM from a 1000X stock solution prepared in absolute ethanol^38^. An equivalent concentration (0.1%) of ethanol was added to the SCONT group. COCs were incubated for 24 h in a humidified atmosphere with 5% CO_2_ at 38.5 °C.

### In vitro fertilization (IVF)

After 24 h of IVM, COCs were washed and transferred to fertilization medium (Fert-TALP medium containing 0.72 U/ml heparin) and 10^6^ motile spermatozoa/ml in four-well plates (40–110 COCs/well). Frozen bull semen from the same ejaculate with proven in vivo and in vitro fertility was acquired from a cattle breeding organisation (CRV, Wirdum, The Netherlands) and used for IVF. Motile spermatozoa were separated by centrifugation (10 min at 971 g and 10 min at 155 g) through a Percoll gradient (90–45% Percoll). COCs were co-incubated with spermatozoa for 20 h in a humified atmosphere with 5% CO_2_ at 38.5 °C. In some replicates, COCs were collected at this stage for denudation and assessment of ATP production rate in the MII oocytes.

### In vitro culture (IVC)

At 20 h after IVF, presumptive zygotes were denuded by vortexing for 3 min in Wash-TALP and washed in droplets of 100 µl Wash-TALP. Presumptive zygotes were then transferred to a 96-well plate in groups of 25 ± 3 in 75 µl of synthetic oviductal fluid (SOF) containing 2% BSA as a carrier of PA. In the PA group, PA was added at a concentration of 230 µM from a 1000X stock solution prepared in absolute ethanol^38^. An equivalent concentration (0.1%) of ethanol was added to the SCONT group. The embryos were incubated in 90% N_2_, 5% CO_2_, 5% O_2_ at 38.5 °C until day 4.7 post fertilization (p.f.) for morula collection, or until the blastocyst stage on day 8 p.f.

At 48 h p.f., embryos were assessed under the inverted light microscope (Olympus CKX41) and classified as not-cleaved, 2-cell, 3-cell, 4 cells or more, or fragmented (cells are asymmetric and/or not uniform in color and density). Also, on day 4.7 p.f. morula rates were counted. Finally, on day 7 and day 8 p.f., blastocyst rates were recorded.

According to the experimental design, zygotes (24 h p.f.), 4-cell embryos (48 h p.f.) and/or morulae (4.7d p.f.) were removed from culture for further analysis. Collected zygotes and morulae were fixed for assessment of global DNA methylation and histone methylation/acetylation, while other zygotes and morulae were snap-frozen for assessment of gene expression. 4-cell embryos were collected in droplets of wash-TALP and used for assessment of MMP or MT ATP production rate. In these replicates, IVC was terminated at the 4-cell or morula stage.

### Global DNA methylation and histone methylation/acetylation

Zygotes and morulae were fixed in 4% paraformaldehyde (PFA) for 15 min and then stored in PBS containing 1 mg/ml polyvinylpyrrolidone (PVP) (PBS-PVP). All embryos were immunostained for 5mC only or double immunostained for H3K9ac and H3K9me2, and imaged as previously described^27,56^ using primary (5mC: rabbit 5mC, Cell Signal, Leiden, The Netherlands—1:1600 dilution; H3K9ac: mouse H3K9ac, Abcam, Cambridge, United Kingdom—1:500 dilution; H3K9me2: rabbit H3K9me2 antibody, Cell Signal – 1:250 dilution) and secondary antibodies (5mC/H3K9me2: goat anti-rabbit FITC secondary antibody, Thermo Fisher Scientific, Leuven, Belgium—1:200 dilution; H3K9ac: goat anti-mouse Texas-Red antibody, Thermo Fisher Scientific—1:200 dilution). Negative controls were incubated in equivalent concentrations of normal rabbit IgG (5mC and H3K9me2) or normal mouse IgG (H3K9ac) instead of the primary antibodies. Stained embryos and negative staining controls were mounted in droplets of DABCO and immediately examined under a Leica SP8 confocal microscope (Leica, Machelen, Belgium) and equipped with white laser source (WLL, Leica) at excitation/emission 488/525 nm (to visualize FITC-labelled 5mC or H3K9me2) and 530/620 nm (to visualize Texas-red labelled H3K9ac). For each embryo, scanned depth was ± 14 µm with 1 µm interval. Using ImageJ software, only the (pro)nuclei were selected and the the gray scale intensity of every channel in each nucleus at each z-stack was quantified using ImageJ software and averaged to generate an average mean gray intensity of 5mC, H3K9ac and H3K9me2 for each embryo. For H3K9me2 in zygotes, the background was high and the intensity of the nuclei is relatively low at this stage. However, we still managed to visualize the pronuclei. See Supplementary Figs. S1 and S2 online for representative images.

### Mitochondrial membrane potential (MMP)

MMP was assessed in 4-cell stage embryos at 48 h p.f. by a fluorescence staining with JC-1 (5,5′,6,6′-tetrachloro-1,1′,3,3′-tetraethyl-benzimidazolyl-carbocyanine iodide, Thermo Fisher Scientific) as described by Komatsu et al.^70^ and Marei et al.^69^. 4-cell stage embryos were incubated in Wash-TALP containing JC-1 (5 μg/mL) (from 1000 × stock solutions in dimethyl sulfoxide) for 30 min at 5% CO_2_, 5% O_2_ at 38.5 °C. Then they were washed and transferred to Wash-TALP droplets under mineral oil in 35 mm glass-bottom dishes. Stained embryos and negative staining controls were immediately examined under a Leica SP8 confocal microscope (Leica) enclosed in a controlled environment (37 °C) and equipped with white laser source (WLL, Leica) at excitation/emission 488/525 nm (to visualize green JC-1 monomers indicating MT with low MMP) and 561/590 nm (for the yellow JC1-aggregates which are formed when MMP is high). Using ImageJ software, only the nuclei were selected and the gray scale intensity of the cells in each channel in the mid-plane and subcortical area of the embryo was quantified. MMP was calculated as a ratio of the gray scale intensity at 590 nm:525 nm. See Supplementary Fig. S3 online for representative images.

### ATP production rate

Preparation and analysis were performed as in Muller et al.^71^. COC’s (after 24 h IVM) were collected in Wash-TALP containing 0.3 mg/ml hyaluronidase to remove cumulus cells. 4-cell embryos were collected in Wash-TALP at 48 h p.f.. Both were then washed in Seahorse analysis medium (Seahorse XF DMEM assay medium with 10 mM Seahorse XF glucose solution, 1 mM Seahorse XF pyruvate solution and 2 mM Seahorse XF glutamine solution) and transferred to a Seahorse XFp Cell Culture Miniplate containing Seahorse analysis medium in pools of 14–20 (MII oocytes) or 10–22 (4-cell embryos). The number of oocytes or embryos per group was fixed in each replicate. Then, the plate was inserted in the Seahorse XFp HS Mini Bioanalyzer after an equilibration step of the sensors to perform the ATP Rate Assay following the manufacturer instructions. Oxygen consumption rate (OCR) is a measure for the concentration of O_2_ and extracellular acidification rate (ECAR) is a parameter for the concentration of H^+^ that are measured in the microchamber. MT ATP production rate is the rate of oxygen consumption that is coupled to ATP production during OXPHOS and can be calculated as the OCR that is inhibited by addition of oligomycin A (ATP synthase inhibitor): OCR_ATP_ (pmol O_2_/min) = OCR (pmol O_2_/min) − OCR_Oligo_ (pmol 0_2_/min). Glycolytic ATP production rate is equivalent to the glycolytic proton efflux rate (PER), which is a measure of ECAR that accounts for the buffer factor and effective volume of the microchamber. Total ATP production rate is calculated as the sum of MT and glycolytic ATP production rate. See Supplementary Fig. S4 online for kinetic graphs of OCR and ECAR.

### Gene expression

Zygotes (pools of 10–20) and morulae (pools of 7–30 depending on the yield and group effects) were washed in droplets of PBS containing 1 mg/mL PVP, transferred in minimal volume to a 1.5 ml vial and immediately snap-frozen in liquid nitrogen. Samples were stored at − 80 °C. RNA extraction was carried out using PicoPure RNA Isolation Kit (Thermo Fisher Scientific) according to manufacturer instructions. RNA samples were DNase-treated and cDNA was synthesized using Sensiscript RT kit (50 ng total RNA/reaction, Qiagen, Hilden, Germany). The quantification of mRNA transcripts was performed by RT-qPCR using SYBR Green. No reverse transcription (NRT) and no template control (NTC) were included as negative controls. After RT-qPCR, the quantification was normalized using the geometric mean of the housekeeping genes 18S ribosomal RNA (18S) and tyrosine 3-monooxygenase/tryptophan 5-monooxygenase activation protein zeta (YWHAZ)^69,72^. Then, the relative expression of each gene was calculated using the 2^−ΔΔCT^ method, as described by Pfaffl^73^. Target genes were chosen based on their importance and expression levels at the zygote and morula stage^74–76^. Also, genes related to MT function and oxidative stress were analysed (Table 3).

**Table 3:** Expression of genes that was examined in zygotes and morulae using qPCR.

Process	Zygote	Morula
DNA (de)methylation	DNMT3b, TET2, TET3	TET1
Histone (de)methylation	EHMT1, EHMT2	EHMT1, KDM3a
Histone (de)acetylation	HAT1, HDAC3	HAT1, HDAC2
MT function	HSPD1, TFAM	HSPD1, TFAM
Oxidative stress	SOD2	SOD2

*DNMT3b* DNA methyltransferase 3 beta, *TET1/2/3* Ten-eleven translocase methylcytosine dioxygenase 1/2/3, *EHMT1/2* Euchromatic histone-lysine *N*-meyhyltransferase ½, *KDM3a* Lysine demethylase 3a, *HAT1* Histone acetyltransferase 1, *HDAC2/3* Histone deacetylase 2/3, *SOD2* Superoxide dismutase 2, *HSPD1* Heat shock protein family D member 1, *TFAM* Transcription factor A, mitochondrial.

Reference sequences of *Bos taurus* from the National Center for Biotechnology Information (NCBI) were used for primer design using the Primer-Blast tool. All primers were exon spanning. See Supplementary Table S1 online for full primer list.

### Statistical analysis

Statistical analyses were performed using IBM Statistics SPSS 28 (for Windows, Chicago, IL, USA). All categorical data (cleavage, morula and blastocyst rates) were compared with binary logistic regression including replicate as a random factor. Numerical data (5mC/H3K9ac/H3K9me2 and JC-1 immunostaining, Seahorse and RT-qPCR) were tested for normality of distribution (Kolmogorov–Smirnov test) and equality of variance (Levene’s test) and the means were compared with linear mixed model. H3K9me2 and JC-1 immunostaining, glycolytic ATP production (Seahorse) and RT-qPCR data were not normally distributed and thus analysed using the non-parametric Kruskal–Wallis test. Interaction between group and IVP replicate effects was tested. If the interaction term was not significant it was left out from the final model. A *P* value of ≤ 0.05 was considered as significant (indicated with a different letter or ‘*’ in the figures) while *P* values between 0.05 and 0.1 were considered as tendencies (indicated with ‘$’ in the figures). Data are expressed as means ± SEM.

### Supplementary Information

Supplementary Information.

## Data Availability

Datasets are deposited in Mendeley Data, 10.17632/8nspk97h5t.1.
