# Supplementary material for: Effect of lipotoxicity on mitochondrial function and epigenetic programming during bovine in vitro embryo production
Source: Sci Rep. 2023 Dec 8;13:21664. doi: 10.1038/s41598-023-49184-0 (PMC10709407; doi:10.1038/s41598-023-49184-0)
Supplement: Supplementary file 1 — Supplementary Information. [file 41598_2023_49184_MOESM1_ESM.pdf]

Supplementary information

Effect of lipotoxicity on mitochondrial function and epigenetic programming during bovine *in vitro* embryo production

Ben Meulders[1,*], Waleed F.A. Marei[1,2], Inne Xhonneux[1], Peter E.J. Bols[1], Jo L.M.R. Leroy[1]

[1]University of Antwerp, Department of Veterinary Sciences, Laboratory of Veterinary Physiology and Biochemistry, Gamete Research Centre, Wilrijk, Belgium.

[2]Cairo University, Department of Theriogenology, Faculty of Veterinary Medicine, Giza, Egypt.

*Ben.Meulders@uantwerp.be

Supplementary Figure S1: Representative confocal images of central z-plane from zygotes after immunostaining for global DNA methylation (5mC) and histone acetylation/methylation (H3K9ac/H3K9me2). Embryos were examined under a SP8 confocal microscope (Leica, Machelen, Belgium) and equipped with white laser source (WLL, Leica) at excitation/emission 488/525 nm (to visualize FITC-labelled 5mC or H3K9me2) and 530/620 nm (to visualize Texas-red labelled H3K9ac). The pronuclei (full arrows) are indicated in the figures. The demarcation of the zygote is indicated with a dotted line.

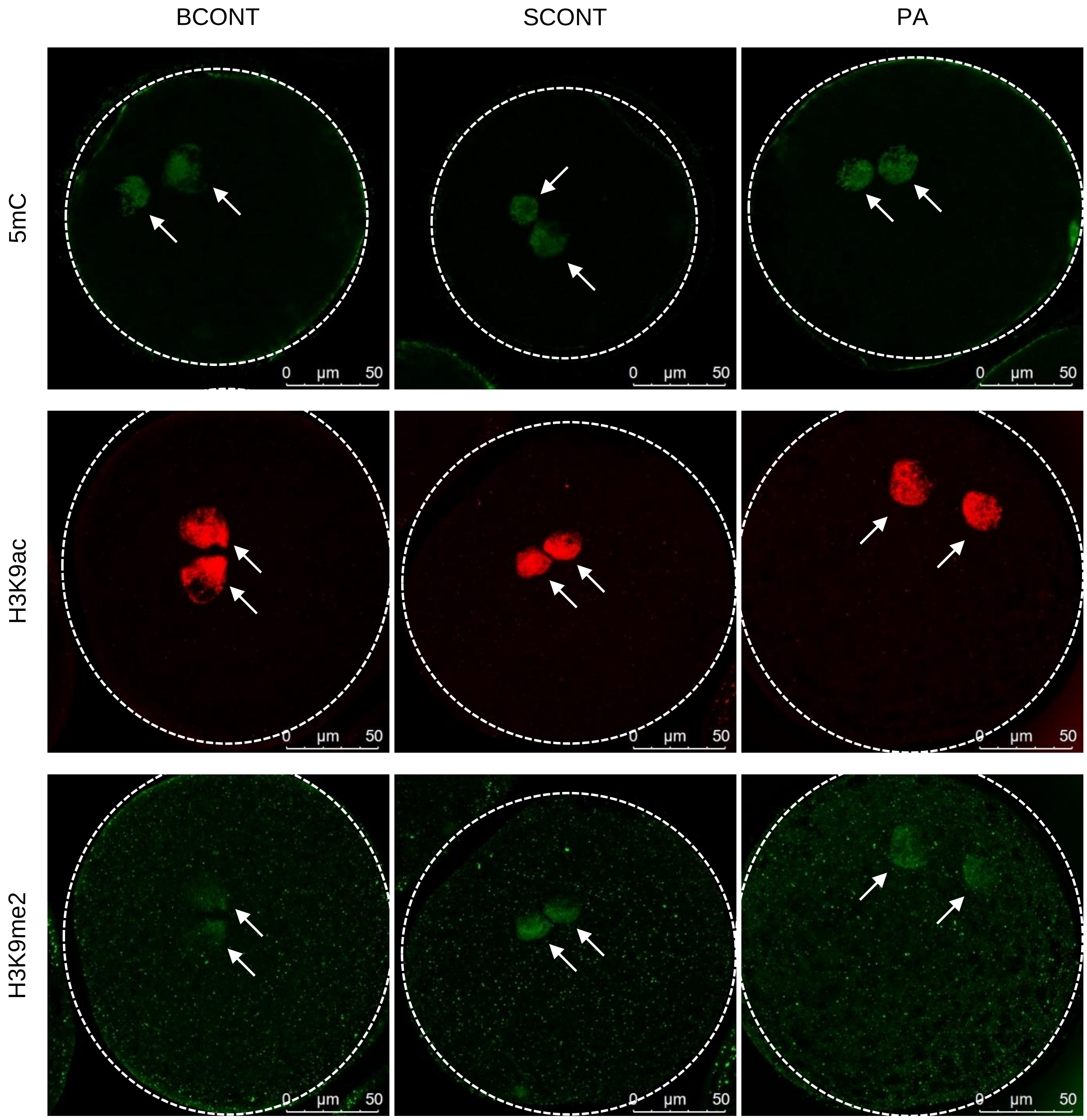

Supplementary Figure S2: Representative confocal images of central z-plane from morulae after immunostaining for global DNA methylation (5mC) and histone acetylation/methylation (H3K9ac/H3K9me2). Embryos were examined under a SP8 confocal microscope (Leica, Machelen, Belgium) and equipped with white laser source (WLL, Leica) at excitation/emission 488/525 nm (to visualize FITC-labelled 5mC or H3K9me2) and 530/620 nm (to visualize Texas-red labelled H3K9ac). The demarcation of the morula is indicated with a dotted line.

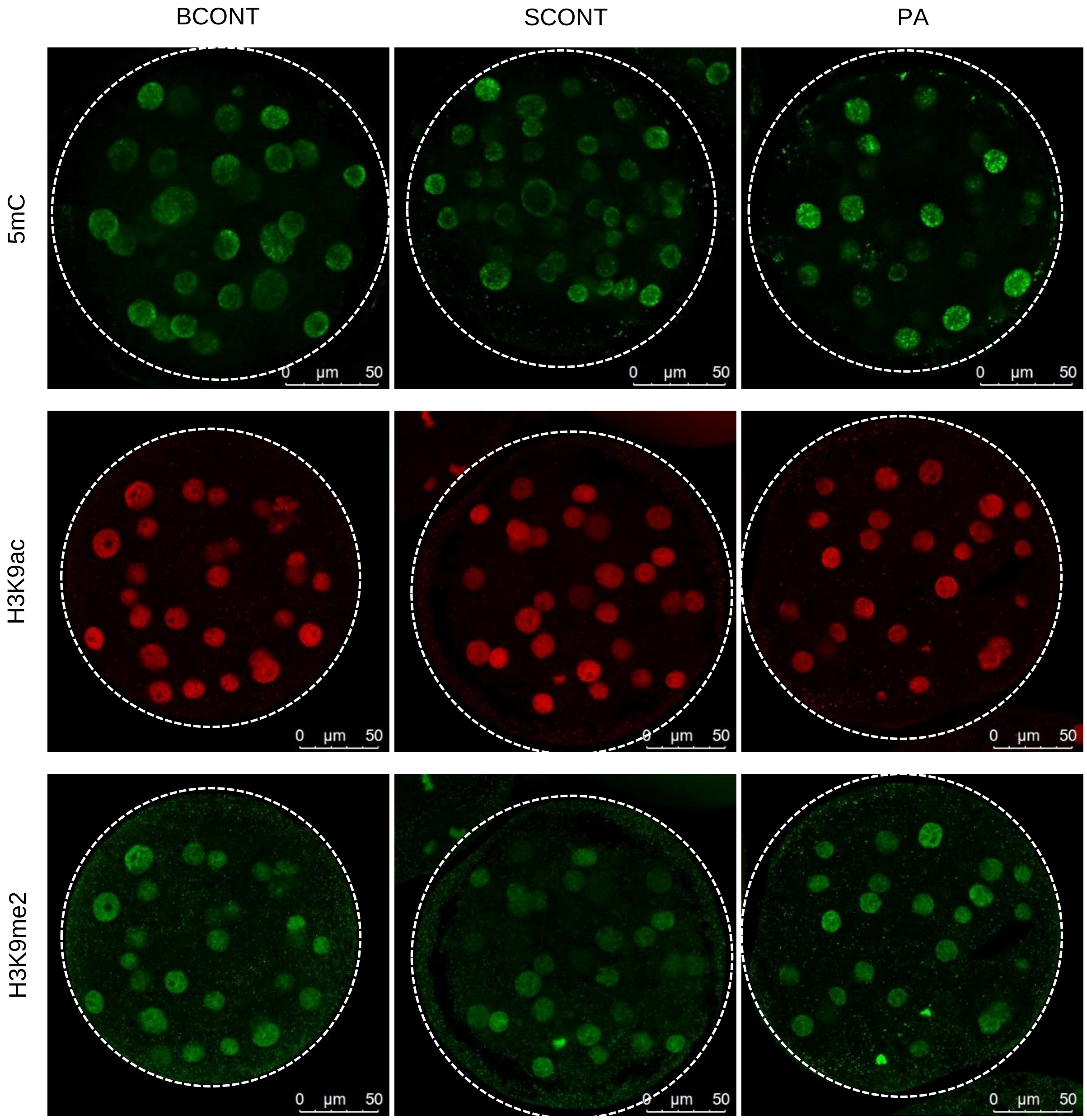

Supplementary Figure S3: Representative confocal images of central z-plane from 4-cell embryos after immunostaining with 5,5',6,6'-tetrachloro-1,1',3,3'-tetraethyl-benzimidazolyl-carbocyanine iodide (JC-1). Embryos were examined under a SP8 confocal microscope (Leica, Machelen, Belgium) and equipped with white laser source (WLL, Leica) at excitation/emission 488/525 nm (to visualize green JC-1 monomers indicating MT with low MMP) and 561/590 nm (for the yellow JC1-aggregates which are formed when MMP is high). For analysis, only the blastomeres were selected based on the channel showing the green JC-1 monomers since the signal outside the embryos originates from the remnants of cumulus cells and their transzonal projections. The demarcation of the 4-cell embryo is indicated with a dotted line.

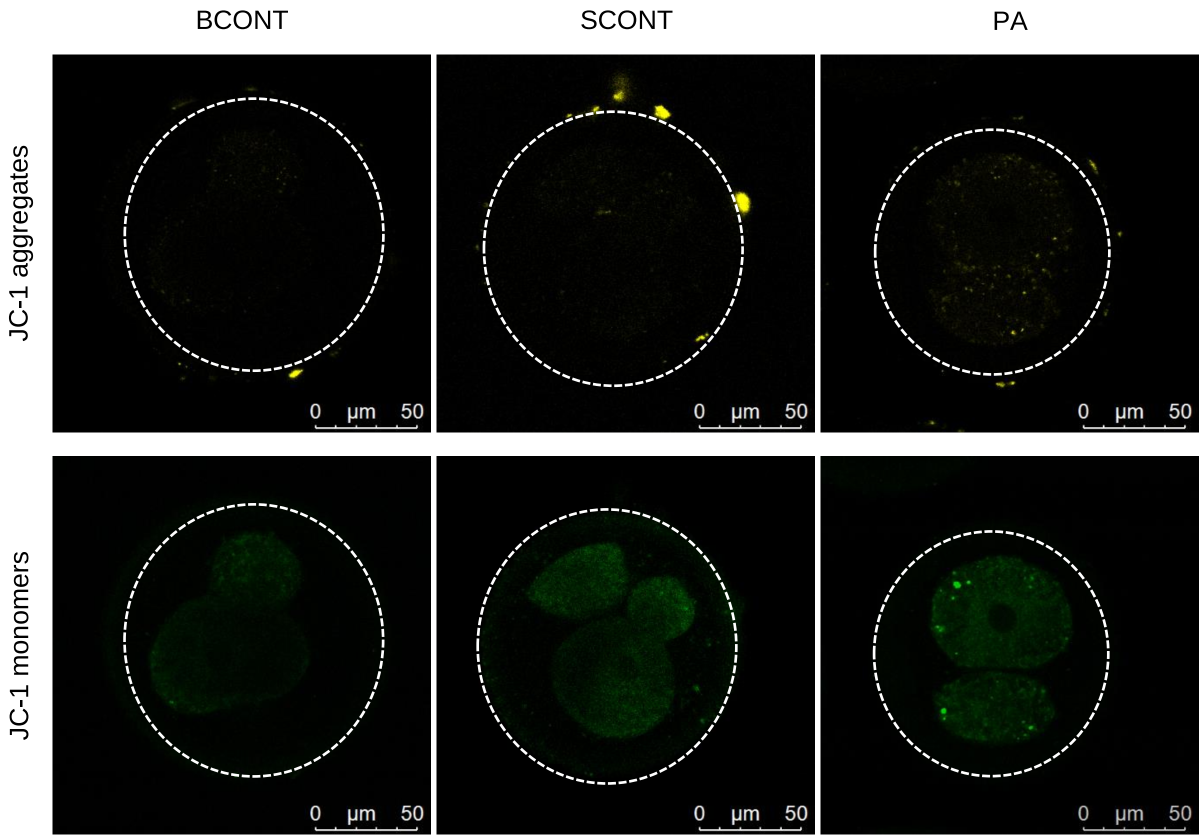

Supplementary Figure S4: Kinetic graphs of the average oxygen consumption rate (OCR) and extracellular acidification rate (ECAR) of all replicates for all treatment groups in (a) oocytes and (b) 4-cell embryos. OCR was measured using the ATP Rate Assay Kit of the Seahorse Xfp Bioanalyzer

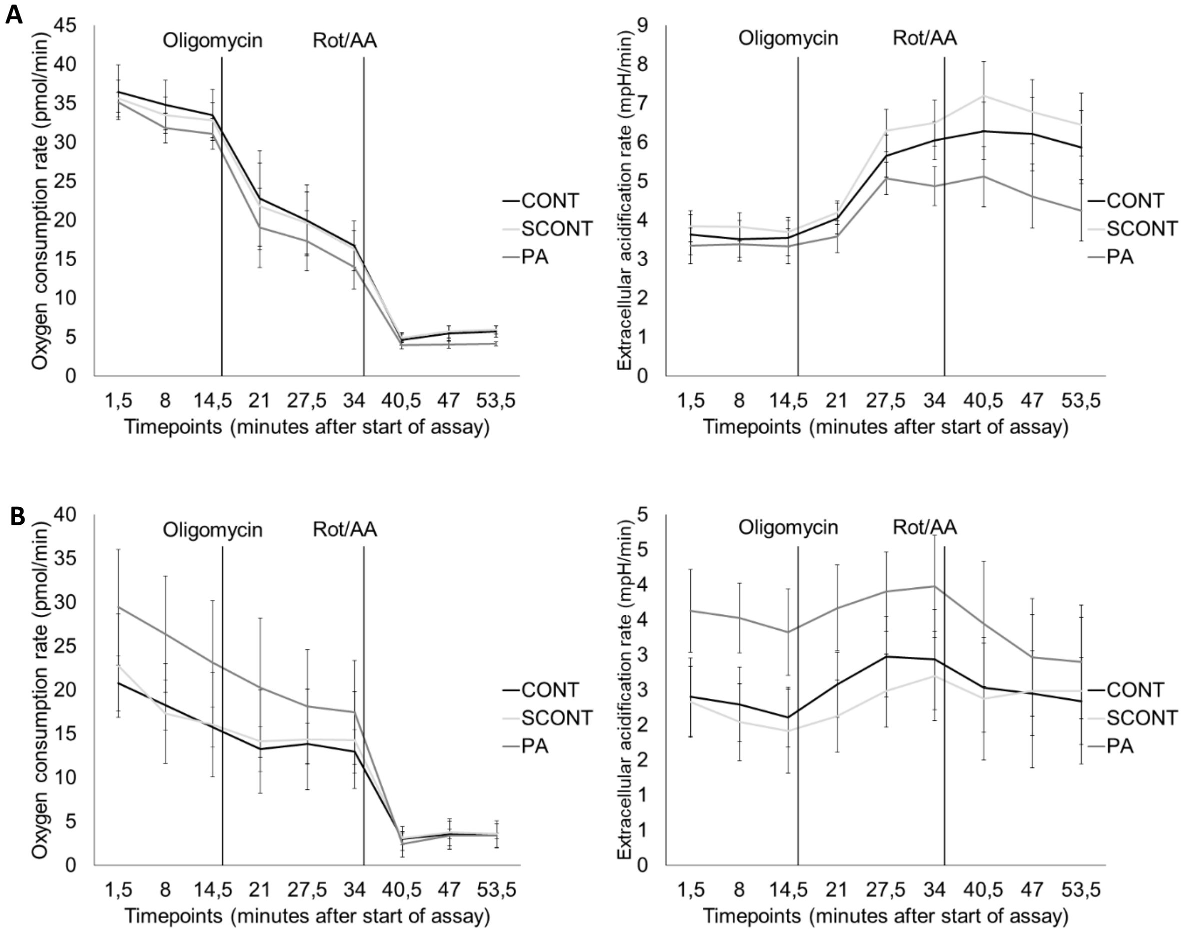

Supplementary table S1: Primer details used for gene expression analysis of zygotes and morulae.

Gene	Primer sequence (5’-3’)	Fragment size (BP)	Genebank Accession NO.	Annealing T (°C)	Reading T (°C)
18S		169	AF176811.1	61.4	76.0
Forward	AGAAACGGCTACCACATCCA				
Reverse	CACCAGACTTGCCCTCCA				
YWHAZ		120	BM446307.1	61.4	76.0
Forward	GCATCCCACAGACTATTTCC				
Reverse	GCAAAGACAATGACAGACCA				
DNMT3b		210	NM_001206502.2	63.3	84.5
Forward	GCGTTACACAGAAGCATATCCAG				
Reverse	CCACATTCTCAAAGAGCCAGAAG				
TET1		283	XM_024986938.1	59.0	79.0
Forward	ATGAAACCGGAACCAATCTGTC				
Reverse	GTGTTAAAGTGCAGACCACTGT				
TET2		156	XM_010805937.3	63.3	70.0
Forward	TGGATACACCTGTCAAGACTCAG				
Reverse	CCTTCTGTCCAAACCTTTCTTCC				
TET3		186	XM_024999364.1	63.3	83.0
Forward	CCTGGAGCATGTACTTCAATGG				
Reverse	CATTGGTCACCTGGTTCTGATAG				
EHMT1		154	NM_001099041.1	63.3	77.0
Forward	CAGCTGCAGTATCTCGGAAG				
Reverse	CAGATCCTTCTCACCCTTGGA				
EHMT2		140	NM_001206263.2	61.4	84.0

Forward	CTGGAGAAACTGCAAGAACAGAG				
Reverse	CTCCGACATACTCGCAAATGAAG				
KDM3a		216	NM_001192872.3	63.3	77.0
Forward	GTGTGACCAAGAAGAAGAAGTCC				
Reverse	CTCTGATCATGAATGGGATCGTG				
HAT1		125	NM_001034347.1	63.3	75.0
Forward	CGGAAATGGCGGGTTTGATTA				
Reverse	CCAGTTTCTTCTCAACTGCACTC				
HDAC2		200	NM_001075146.1	63.3	75.0
Forward	CTGGAACAGGAGACTTAAGGGATA				
Reverse	ACCAAGTCTATCACCAGATAGGG				
HDAC3		262	NM_001206243.1	63.3	83
Forward	GCTGCTGGACATATGAGACATC				
Reverse	TCATCAGTCCTGTCGTAGGTTAG				
SOD2		181	NM_201527.2	61.0	78.0
Forward	TGCAAGGAACAACAGGTCTTATC				
Reverse	CTCAGTGTAAGGCTGACGGTT				
TFAM		293	NM_001034016.2	61.4	76.0
Forward	GCCAAGCTATGGAGGGAACT				
Reverse	AGCTTTACCTGTGATGTGCCA				
HSPD1		159	NM_01166608.1	63.0	78.0
Forward	CTACTGTACTGGCACGCTCT				
Reverse	CAATCTCTTCGGGGGTTGTC				
